# Scurvy in the Modern World: Extinct or Not?

**DOI:** 10.7759/cureus.22622

**Published:** 2022-02-26

**Authors:** FNU Amisha, Sai Nikhila Ghanta, Amudha Kumar, Tyler Fugere, Paras Malik, Sunilkumar Kakadia

**Affiliations:** 1 Internal Medicine, University of Arkansas for Medical Sciences, Little Rock, USA; 2 Internal Medicine, Jacobi Medical Center-Albert Einstein College of Medicine, New York, USA; 3 Hematology and Medical Oncology, University of Arkansas for Medical Sciences, Little Rock, USA

**Keywords:** rare case report, case report, vitamin c, ascorbic acid deficiency, ascorbic acid, vitamin c deficiency, scurvy

## Abstract

Scurvy is a nutritional disorder caused by vitamin C deficiency. It was a notorious disease in the ancient world, especially among the sailors, and is of rare occurrence in contemporary, developed countries due to increased access and advancement in nutrition services. Scurvy primarily affects the skin and soft tissue, presenting with a myriad of clinical manifestations ranging from musculoskeletal to bleeding-related complaints and even sudden death in later stages. In this article, we present the case of an elderly female with scurvy-related weakness and gait instability leading to mechanical falls, easy bruising, fatigue, and petechial rash. She had improvement in her constitutional symptoms after the initiation of vitamin C supplements. This case reinforces the need to consider scurvy as one of the differentials for petechial rash and easy bruising apart from bleeding diathesis and vasculitis in the contemporary world, especially in at-risk populations.

## Introduction

Vitamin C aka ascorbic acid is a water-soluble vitamin that is the enolic form of alpha-ketolactone. It is usually absorbed in the distal small intestine via adenosine triphosphate (ATP)-dependent active transportation and tends to concentrate in the pituitary, adrenal gland, brain, white blood cells, and eyes for storage and utilization [1]. Most animals, except for apes (including humans), rodents, bats, and monkeys, synthesize ascorbic acid in their own bodies. The recommended dietary allowance (RDA) of ascorbic acid is 90 mg/day for men and 75 mg/day for women, which will provide for a body pool of 1500 mg and maintain ascorbate reserves for one to three months [2]. Pregnant females and elderly people have increased requirements of up to 120 mg/dl total and smokers up to 35 mg/dl above the RDA. Scurvy can occur due to decreased dietary intake or impaired gastrointestinal vitamin C absorption.

Scurvy initially presents with musculoskeletal complaints in the form of weakness, arthralgias, myalgias, and fatigue, especially in the upper legs and thighs. This is due to the defective osteoid matrix, increased bone resorption, and secondary edema. The subsequent stages involve depression, follicular hyperkeratosis, poor wound healing, corkscrew hair, and swollen gums. Later stages involve hemorrhagic features like ecchymoses, perifollicular hemorrhage, petechiae, hemarthroses, hemopericardium, etc., to infection or sudden death [3-4]. Imaging findings include metaphyseal head lucency, soft-tissue edema, and periosteal reaction. With appropriate treatment, constitutional symptoms can improve as early as 24 hours, skin manifestations like ecchymosis or bruising resolve within a few days, whereas musculoskeletal symptoms might take as long as two weeks for recovery [5]. Herein, we present an interesting case of an elderly female with classic symptoms and signs of this ancient disease. Early diagnosis of scurvy is the key for clinical improvement, prevention of complications, and reduction of additional health care costs by avoiding unnecessary medical tests.

## Case presentation

**Figure 1:**
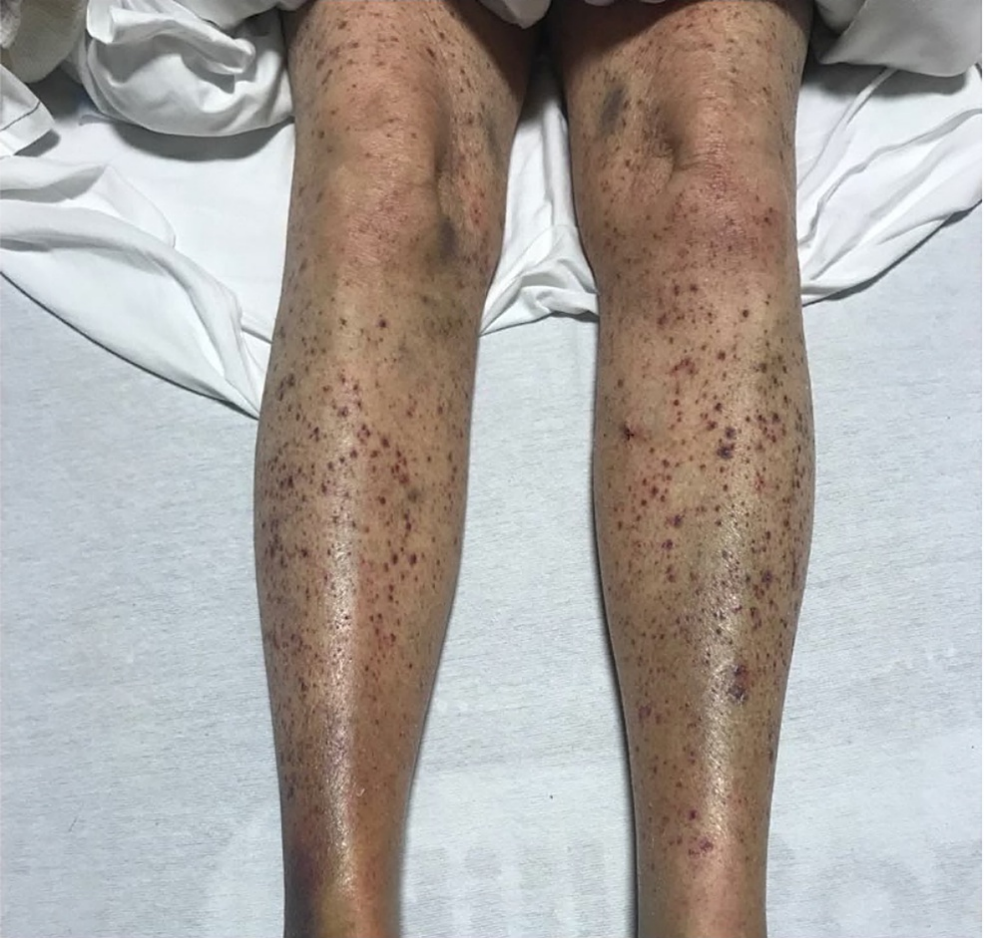
Image depicting the non-palpable petechia on bilateral lower extremities and blue ecchymosis on bilateral ankles

**Figure 2:**
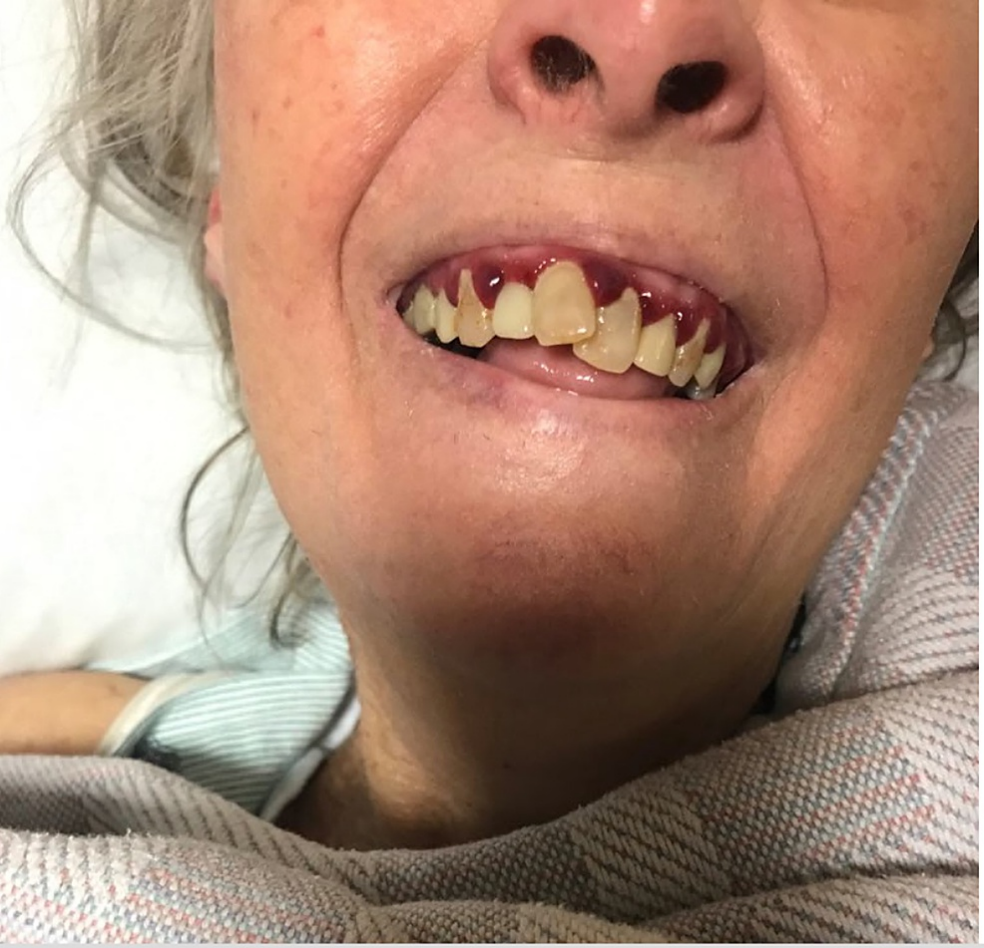
Image depicting gingival redness and bruising

A 72-year-old white female with a history of esophageal stricture and multiple dilatations, mild cognitive impairment, and left breast adenocarcinoma status post lumpectomy and chemoradiation, in remission for 13 years, was admitted with a bilateral ankle injury after a mechanical fall two weeks prior due to generalized weakness and difficulty walking. Further history revealed decreased appetite, difficulty swallowing, fatigue, malaise, easy bruising, and 16 pounds weight loss over the last three months. She had good socio-economic status, and her diet mainly consisted of canned food, ready-to-eat meals, tea, toast, or diet soda with a little vegetable or fruit intake. She denied chills, night sweats, cough, chest discomfort, haematochezia, melena, hematemesis, alcohol, smoking or illicit drug use, herbal supplements, non-steroidal anti-inflammatory drugs (NSAIDs), and anticoagulant or antiplatelet drug use. On physical examination, she had bilateral lower extremity non-palpable petechial rash, multiple large blue-red painful ecchymosis of varying age over bilateral thighs and both ankles, and gingival inflammation (Figures 1-2).

Initial laboratory investigations are described in Table 1. Major differential diagnoses included vasculitis (viral, bacterial, leukocytoclastic, or malignancy-related) or vitamin deficiency. Laboratory workup, including peripheral blood smear, antinuclear and antineutrophil cytoplasmic antibody, serum, and urine protein electrophoresis, cryoglobulins and complements, hepatitis, and human immunodeficiency virus panel were unremarkable. Magnetic resonance imaging of bilateral ankles revealed multiple intraosseous hemorrhages with diffuse intramuscular and subcutaneous edema. A punch biopsy of the left lower leg showed corkscrew hair with perifollicular hemorrhage and perivascular inflammation with no evidence of microthrombi. Her serum ascorbic acid level was found to be less than 0.05 mg/dl (ref: 0.2- 2 mg/dl), and a diagnosis of scurvy was made. Additional nutritional workup showed decreased serum levels of vitamin A (0.17, ref: 0.3-1.3 mg/L), vitamin E (4.9, ref: 5.5-18 mg/L), vitamin B1 (54, ref: 70-180 nmol/L ), vitamin B12 (249, ref: 118-914 pg/ml), and vitamin B9 (3.78, ref: 5.9->22.3 ng/ml). She was initially started on 500 mg ascorbic acid daily along with multivitamins with significant improvement in her constitutional symptoms within 24 hours. She was lost to follow-up after discharge from the hospital.

**Table 1:** Initial laboratory investigations on admission BUN: blood urea nitrogen; AST: aspartate transaminase; ALT: alanine aminotransferase; GGT: gamma-glutamyl transferase; LDH: lactate dehydrogenase

Investigation	Value	Reference range
Hemogram		
Hemoglobin (g/dl)	9.9	13-17
WBC K/uL (N/L/M/E/B %)	3 (50/37/8.8/3.1/0.8)	3.6-9.5 ( 35-65/23-50/4.6-12/0.5-6.5/0.1-1.1)
Platelets (K/Ul)	230	150-450
Mean corpuscular volume (fl)	97	80-100
Mean corpuscular hemoglobin (pg)	33	26-33
Mean corpuscular hemoglobin concentration (g/dL)	34	32-36
Absolute neutrophil count	1.93	>1500 cells/ul
Renal chemistry		
Na/K/Cl/CO2 (mmol/L)	135/4.8/108/19	135-145/3.5-5.1/98-107/22-32
BUN/Cr (mg/dL)	10/0.7	6-20/0.6-1.3
Liver function test		
AST/ALT (IU/L)	18/11	15-41/4-45
GGT (IU/L)	12	7-50
LDH (IU/L)	250	100-248
Alkaline phosphatase (IU/L)	78	32-91
Bilirubin, Total (mg/dL)	1.8	0.2-1.2
Miscellaneous		
Retic count	1.2	0.5-2.5%
Ferritin	209	10-300 ng/dl
Total iron-binding capacity	315	250-425 ug/dl
Iron saturation	5	15-50 %
Iron	16	28-100 U/L
Erythrocyte sedimentation rate	66	0-30 mm/hr
C-reactive protein	37.60	< 10 mg/dl
Prothrombin time/international normalized ratio	14/1.2	10.2-12.9 sec/ 0.9-1.1
Activated partial thromboplastin clotting time	30.3	25.1-36.5 sec
Rheumatoid factor	79.8	< 12.5 IU/ml

## Discussion

Vitamin C is vital for wound healing, functioning of the immune system, production of neurotransmitters like dopamine and prostaglandins, nitric oxide synthesis, and osteoblast and fibroblast growth. Recent research has associated vitamin C with atherosclerotic cardiovascular disease by inhibiting low-density lipoprotein oxidation, resulting in improved arterial stiffness, lipid profile, and endothelial function [6]. Vitamin C is essential for maintaining the triple helical structure of collagen via the hydroxylation of proline and lysine residues. Collagen is required for the integrity of blood vessels and disruption of collagen results in weak and leaky vessels with an abnormal bruising and petechial rash if deficient as in our patient [7]. In addition, vitamin C, through its various enzymatic and non-enzymatic biochemical reactions, acts as an electron donor (reducing agent) for vitamin E, folic acid, iron, and copper production. This can explain the multiple vitamin deficiencies in our patient apart from malnutrition.

In the human diet, 90% of vitamin C comes from citrus fruit and vegetables like sweet peppers, broccoli, cauliflower, and tomatoes. Thermal treatment can decrease the vitamin C content of food. It takes 40-90 days of sustained hypovitaminosis C for overt scurvy to develop, but clinical manifestations can appear within 30 days of dietary insufficiency of vitamin C. While plasma concentration is reflective of recent intake, leukocyte vitamin C concentration is a better indicator of total body stores [8]. Clinical scurvy occurs when the total body pool is less than 300 mg and the plasma level is <0.2 mg/dl (11 umol/L) [6]. Our patient had a plasma ascorbic level <0.05 mg/dl and repeat levels were not drawn after supplementation because she was lost to follow up.

While the global prevalence varies, the estimated prevalence of vitamin C deficiency in the United States (US) is 7.1% [9]. Since the 1970s, the American diet has changed a lot, with increased intake of potatoes, pizzas, and carbonated drinks, and less intake of fruits, vegetables, and dairy products, which has led to a resurgence of nutritional disorders like scurvy and increased prevalence of metabolic disorders like obesity and diabetes [10]. This is attributable to busy work schedules, convenience, inadequate social support, and a sedentary lifestyle. High-risk groups for scurvy in the modern world include people prone to malnutrition - psychiatric illness, eating disorders, alcoholism; limited food access - elderly living alone, homelessness, COVID-19 pandemic; or malabsorptive states - gastric bypass, celiac disease, hemodialysis [11]. A cultural change towards better food choices, availability of healthier ready-to-eat meals, food fortification, and early initiation of nutritional supplements for high-risk populations may be considered.

## Conclusions

Scurvy is a great mimic, with manifestations ranging from weakness to sudden cardiac death or death from infection. The rarity and wide clinical features contribute to delay in diagnosis. We believe it is important for internists to consider this as a differential, especially in certain high-risk groups. Keeping in mind the good safety profile and low cost, empiric treatment with vitamin C should be done as soon as possible in suspected patients.

## References

[1] Schorah CJ (1992). The transport of vitamin C and effects of disease. Proc Nutr Soc.

[2] Institute of Medicine (US) Panel on Dietary Antioxidants and Related Compounds (2000). Dietary Reference Intakes for Vitamin C, Vitamin E, Selenium, and Carotenoids. https://www.nap.edu/read/9810/chapter/7..

[3] Hirschmann J, Raugi G (1999). Adult scurvy. J Am Acad Dermatol.

[4] Wijkmans RA, Talsma K (2016). Modern scurvy. J Surg Case Rep.

[5] Smith A, Di Primio G, Humphrey-Murto S (2011). Scurvy in the developed world. CMAJ.

[6] Moser MA, Chun OK (2016). Vitamin C and heart health: a review based on findings from epidemiologic studies. Int J Mol Sci.

[7] DePhillipo NN, Aman ZS, Kennedy MI, Begley JP, Moatshe G, LaPrade RF (2018). Efficacy of vitamin C supplementation on collagen synthesis and oxidative stress after musculoskeletal injuries: a systematic review. Orthop J Sports Med.

[8] Maxfield L, Crane JS (2021). Vitamin C Deficiency. StatPearls [Internet]. Treasure Island (FL.

[9] Schleicher RL, Carroll MD, Ford ES, Lacher DA (2009). Serum vitamin C and the prevalence of vitamin C deficiency in the United States: 2003-2004 National Health and Nutrition Examination Survey (NHANES). Am J Clin Nutr.

[10] Paeratakul S, Ferdinand DP, Champagne CM, Ryan DH, Bray GA (2003). Fast-food consumption among US adults and children: dietary and nutrient intake profile. J Am Diet Assoc.

[11] Monget AL, Galan P, Preziosi P (1996). Micronutrient status in elderly people. Geriatrie/Min. Vit. Aux Network. Int J Vitam Nutr Res.

